# Crystal Structure and Thermal Properties of Double-Complex Salts [M^1^(NH_3_)_6_][M^2^(C_2_O_4_)_3_] (M^1^, M^2^ = Co, Rh) and K_3_[Rh(NH_3_)_6_][Rh(C_2_O_4_)_3_]_2_∙6H_2_O

**DOI:** 10.3390/ijms241512279

**Published:** 2023-07-31

**Authors:** Pavel Smirnov, Evgeny Filatov, Natalia Kuratieva, Pavel Plyusnin, Sergey Korenev

**Affiliations:** 1 Nikolaev Institute of Inorganic Chemistry, Siberian Branch, Russian Academy of Sciences, 630090 Novosibirsk, Russia; 2 Department of Natural Sciences, Novosibirsk State University, 630090 Novosibirsk, Russia

**Keywords:** complexes of cobalt and rhodium, crystal structure of complex salt, thermolysis, solid solution of metals

## Abstract

Here, seven new double-complex salts, [M^1^(NH_3_)_6_][M^2^(C_2_O_4_)_3_] (M^1^, M^2^ = Co, Rh) and K_3_[Rh(NH_3_)_6_][Rh(C_2_O_4_)_3_]_2_∙6H_2_O types, are synthesised. The crystal structure and composition of DCS (double-complex salts) are studied by SCXRD, XRD, CHN and IR methods. The complex salts of the [M^1^(NH_3_)_6_][M^2^(C_2_O_4_)_3_] (M^1^, M^2^ = Co, Rh) type can be crystallised both as a crystalline hydrate [M^1^(NH_3_)_6_][M^2^(C_2_O_4_)_3_]·3H_2_O (sp. gr. *P*-3) and as an anhydrous complex (sp. gr. *P*-1) depending on the synthesis conditions. The process of [Rh(NH_3_)_6_][Rh(C_2_O_4_)_3_] formation is significantly dependent on the synthesis temperature. At room temperature, a mixture is formed comprising [Rh(NH_3_)_6_][Rh(C_2_O_4_)_3_] and K_3_[Rh(NH_3_)_6_][Rh(C_2_O_4_)_3_]_2_∙6H_2_O, while the [Rh(NH_3_)_6_][Rh(C_2_O_4_)_3_] target product crystallises at elevated temperatures. The thermal behaviour of double-complex salts is studied by the STA, EGA-MS, IR and XRD methods. The complete decomposition of complex salts in helium and hydrogen atmospheres resulting in metals or Co_x_Rh_1−x_ solid solutions is achieved at temperatures of 320–450 °C.

## 1. Introduction

The platinum group metals are widely known to exhibit high catalytic activity in various chemical reactions. Thus, rhodium has found wide application as a catalyst, for example, a heterogeneous rhodium catalyst exhibits high catalytic activity, as well as excellent resistance to coking in the reaction of dry methane reforming [1,2]. However, due to the high cost of rhodium, its use on an industrial scale is limited [3]. One of the ways to solve this problem is to add base metals (Ni, Co, Fe) to rhodium. The catalyst containing rhodium and a base metal has high catalytic activity and stability due to the formation of a synergistic effect between these metals [4,5,6]. A large number of works are devoted to the study of the activity of alloys or solid solutions of cobalt and rhodium of different compositions, which have good catalytic properties, especially in organic synthesis reactions. Thus, bimetallic particles of Co_0.5_Rh_0.5_, obtained from Co_2_Rh_2_(CO)_12_, have demonstrated high catalytic activity in the amino-carbonylation reaction of alkynes in the presence of CO and amines [7]. In [8], the catalytic activity of the Co_x_Rh_100−x_@SILP system (SILP—supported ionic liquid phase) in the hydrogenation reaction of substituted aromatic compounds has been investigated. In particular, it is noted that when the rhodium content in the sample is 75 at.% or more, the catalyst has a typical activity for metallic Rh and is able to completely hydrogenate aromatic substrates. The increase of the cobalt content in the alloy (30–80 at.%) results in a decrease in the activity in the arene hydrogenation, accompanied by an increase in the activity in the C=O bond hydrogenation. Co_30_Rh_70_@SILP and Co_25_Rh_75_@SILP samples are highly active, selective and stable with respect to hydrogenation of benzylideneacetone and various bicyclic heteroaromatic compounds to the corresponding partially and fully saturated products. These works demonstrate the prospects of using solid solutions (alloys) in the Co-Rh system as catalytically active materials.

One promising approach for producing bimetallic nanoparticles is the thermal decomposition of complex salts of DCS precursors in reducing or inert atmospheres. At the same time, carbon- or nitrogen-containing ligands (for example, oxalate, ethylenediamine, ammonia) are selected for DCS production since these ligands have high reducing properties and their thermal decomposition products are highly volatile compounds. Metal atoms in DCS are mixed at the molecular level, which together with a sufficiently low decomposition temperature (ca. 400 °C) makes it possible to form metastable metal solid solutions as a result of thermolysis [9]. Thus, [PdEn_2_]_3_[Rh(C_2_O_4_)_3_]_2_ and [Rh(NH_3_)_6_]_2_[Pd(C_2_O_4_)_2_]_3_ were used to obtain bimetallic Pd-Rh catalysts [10]. The choice of these precursors as starting compounds is associated with the ability of oxalate ions and ammonia molecules to reduce platinum metals to a zero-valent state at sufficiently low temperatures. The thermal decomposition of [Pd(NH_3_)_4_][Pd(NH_3_)_3_NO_2_][Rh(C_2_O_4_)_3_]·H_2_O has been described for inert and reducing atmospheres [11]. The temperature of complete decomposition of that complex compound to metals in the reducing atmosphere is only 275 °C, which demonstrates the prospect of using this precursor to obtain metastable metal alloys in the Pd-Rh system.

The complex salts of the type [M^1^(NH_3_)_6_][M^2^(C_2_O_4_)_3_], M^1^ = Co, Ir, M^2^ = Co, Ir, Fe, Cr have been sufficiently studied over the past 15 years [12,13,14]. Structures and thermal properties in different atmospheres have been investigated for these compounds. The total decomposition temperature of the complexes of the type [M^1^(NH_3_)_6_][M^2^(C_2_O_4_)_3_], M^1^, M^2^ = Co, Ir, Fe, Cr in the atmosphere of hydrogen and helium is 300–400 °C, wherein the final product in all cases is a bimetallic nano-powder of the corresponding metals. The decomposition of these salts in the oxygen atmosphere leads to the formation of oxide systems.

The [Ir(NH_3_)_6_][Ir(C_2_O_4_)_3_] and [Co(NH_3_)_6_][Co(C_2_O_4_)_3_]·3H_2_O have been previously studied [14]. Iridium salt has been shown to crystallise in the *P*-1 space group, while the trihydrate cobalt salt crystallises in the *P*-3 space group. Wherein, [Co(NH_3_)_6_][Co(C_2_O_4_)_3_]·3H_2_O, while standing in the air, loses water, turning into anhydrous salt isostructural to [Ir(NH_3_)_6_][Ir(C_2_O_4_)_3_]. When these compounds are thermolyzed, finely dispersed Co and Ir metals are formed in the reducing atmosphere, respectively, while Ir and (Co + CoO) mixtures are formed in the inert atmosphere.

The aim of this work was the synthesis of DCS [Rh(NH_3_)_6_][Rh(C_2_O_4_)_3_]·3H_2_O, [Rh(NH_3_)_6_][Co(C_2_O_4_)_3_]·3H_2_O, [Co(NH_3_)_6_][Rh(C_2_O_4_)_3_]·3H_2_O, [Rh(NH_3_)_6_][Rh(C_2_O_4_)_3_], [Rh(NH_3_)_6_][Co(C_2_O_4_)_3_], [Co(NH_3_)_6_][Rh(C_2_O_4_)_3_] and K_3_[Rh(NH_3_)_6_][Rh(C_2_O_4_)_3_]_2_·6H_2_O, their characterisation by means of physicochemical methods (XRD, IR spectroscopy and elemental analysis) as well as the study of the thermal decomposition of these DCS in inert and reducing atmospheres. The synthesis of isostructural boundary compounds makes it possible to obtain a continuous series of solid solutions based on DCSs and, accordingly, metal solid solutions.

## 2. Results and Discussion

### 2.1. General Information about DCS

Due to the low solubility of the studied salts, the synthesis was carried out by mixing aqueous solutions of the initial compounds. In this case, a finely crystalline precipitate of composition [M(NH_3_)_6_][M(C_2_O_4_)_3_] or [M(NH_3_)_6_][M(C_2_O_4_)_3_]·3H_2_O was formed.

Compounds in the anionic part, [Rh(C_2_O_4_)_3_]^3−^, were yellow and photostable. Compounds in the anionic part, [Co(C_2_O_4_)_3_]^3−^, were green and photo-decomposed to form a pink substance on the surface of the sample. Most likely, Co(III) was restored to Co(II), and CoC_2_O_4_ was formed.

### 2.2. The Features of [Rh(NH_3_)_6_][Rh(C_2_O_4_)_3_] Synthesis

**Figure 1:**
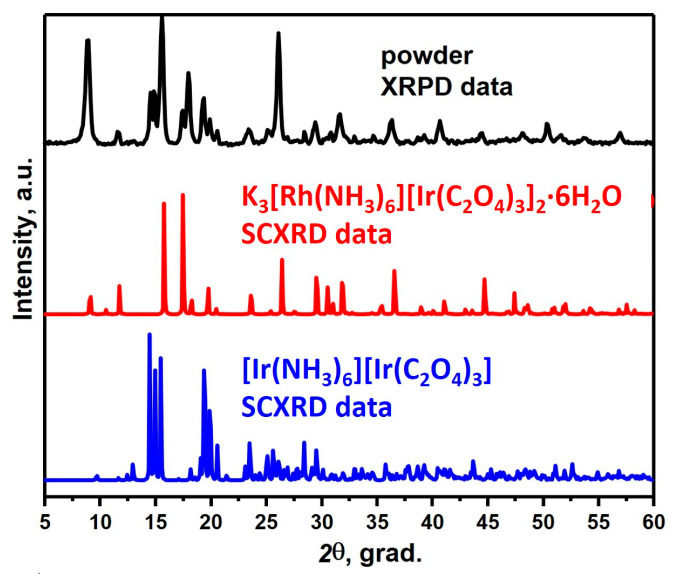
The comparison of XRPD patterns of the obtained two-phase mixture with monocrystalline data for [Ir(NH_3_)_6_][Ir(C_2_O_4_)_3_] and K_3_[Rh(NH_3_)_6_][Rh(C_2_O_4_)_3_]_2_∙6H_2_O.

When synthesising [Rh(NH_3_)_6_][Rh(C_2_O_4_)_3_] from an aqueous solution at room temperature, a precipitate was deposited, which, according to XRD, is a mixture of two phases (Figure 1). The first phase was isostructural to [Ir(NH_3_)_6_][Ir(C_2_O_4_)_3_] with space group *P*-1 [14], but with a shift of reflexes towards smaller angles, while the composition of the second phase was unknown. To determine the composition of the second phase, the mother liquor was evaporated in the air, while two kinds of crystals were obtained from the solution: the first one, according to XRD data, was [Rh(NH_3_)_6_][Rh(C_2_O_4_)_3_]·3H_2_O (sp. gr. *P*-3), and the second was K_3_[Rh(NH_3_)_6_][Rh(C_2_O_4_)_3_]_2_·6H_2_O (sp. gr. *R*-3).

To further study the DCS deposition process, the solubilities of [Rh(NH_3_)_6_][Rh(C_2_O_4_)_3_] and K_3_[Rh(NH_3_)_6_][Rh(C_2_O_4_)_3_]_2_·6H_2_O complex compounds were estimated. They were 1 × 10^−3^–2 × 10^−3^ M and 2 × 10^−3^–3 × 10^−3^ M, respectively. The quite close solubility values indicate that the salts will precipitate together at room temperature. When heated, most likely, the solubility of K_3_[Rh(NH_3_)_6_][Rh(C_2_O_4_)_3_]_2_·6H_2_O increases, so the K_3_[Rh(NH_3_)_6_][Rh(C_2_O_4_)_3_]_2_·6H_2_O phase will dissolve in water, and upon further cooling, the main product would have already precipitated in the form of [Rh(NH_3_)_6_][Rh(C_2_O_4_)_3_].

### 2.3. Crystal Structures of Complex Compounds

#### 2.3.1. The Crystal Structure of [Rh(NH_3_)_6_][Co(C_2_O_4_)_3_]

The structure is an island, and it consists of isolated [Rh(NH_3_)_6_]^3+^ cations and [Co(C_2_O_4_)_3_]^3−^ anions, both located in the common positions. [Rh(NH_3_)_6_]^3+^ complex ions are octahedral. The Rh-N bond lengths are in the range of 2.052(2) to 2.087(2) Å, while the average length of 2.067(7) Å corresponds to the same ions in other structures [15,16]. The N-Rh-N angles are in the range of 88.00(10) to 92.07(11)°, which corresponds to octahedral angles with a maximum deviation of 2.1°. Cations are located in the common positions.

The [Co(C_2_O_4_)_3_]^3−^ complex ions have an airscrew-like form. The Co-O bond lengths are in the range of 1.889(2) to 1.900(2) Å, with an average distance of 1.894(2) Å. The chelate O-Co-O angles are in the range of 86.21(8) to 88.26(9)°. These geometric parameters of the anion correspond to those of the same anions in other structures [12,13,14] formed in the inert atmosphere.

**Figure 2:**
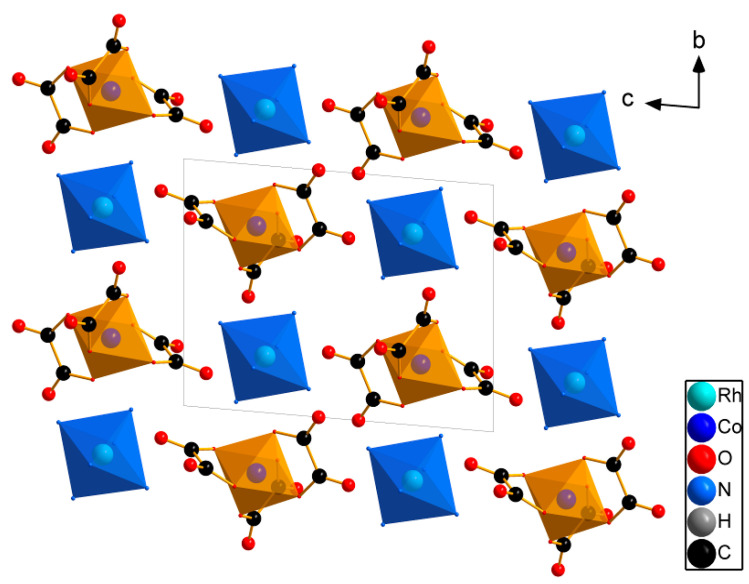
The structure view for [Rh(NH_3_)_6_][Co(C_2_O_4_)_3_]. H atoms are omitted for clarity.

Each complex ion is surrounded by eight neighbouring counter-ion complex species. The Co-Rh distances between the adjacent complex ions range from 5.1143(4) to 7.6708(5) Å. [Rh(NH_3_)_6_]^3+^ and [Co(C_2_O_4_)_3_]^3−^ are hydrogen bonded. This packing crystallises in the structural mode, as in CsCl (Figure 2).

#### 2.3.2. The Crystal Structure of Compounds with *P*-3 Space Group

The crystalline hydrates [Rh(NH_3_)_6_][Rh(C_2_O_4_)_3_]·3H_2_O, [Rh(NH_3_)_6_][Co(C_2_O_4_)_3_]·3H_2_O and [Co(NH_3_)_6_][Rh(C_2_O_4_)_3_]·3H_2_O, with the *P*-3 space group, were crystallised using the slow diffusion of the agar–agar.

The structures are isolated, consisting of separate [M^1^(NH_3_)_6_]^3+^ cations and [M^2^(C_2_O_4_)_3_]^3−^ anions, where M^1^, M^2^ = Co, Rh, and water molecules. The ranges for distances with the average M^1^–N, M^2^–O and valent angles L-M-L are listed in Table 1. Both cations and anions have octahedral coordination spheres. The centres of ions are located on symmetry axis 3 or at the special point of −3.

[M_2_(C_2_O_4_)_3_]^3−^ complex ions are propeller-like. Anions are located on axis 3. For the [Co(NH_3_)_6_][Rh(C_2_O_4_)_3_]∙3H_2_O structure, the positional disordering was found for one anionic [Rh(C_2_O_4_)_3_]^3−^ complex located in the (0, 0, 0), at the special point of −3. This disorder model could be described as the pair of stereoisomers. Therefore, the c parameter for this complex was larger, as compared to [Rh(NH_3_)_6_][Rh(C_2_O_4_)_3_]·3H_2_O and [Rh(NH_3_)_6_][Co(C_2_O_4_)_3_]·3H_2_O.

**Figure 3:**
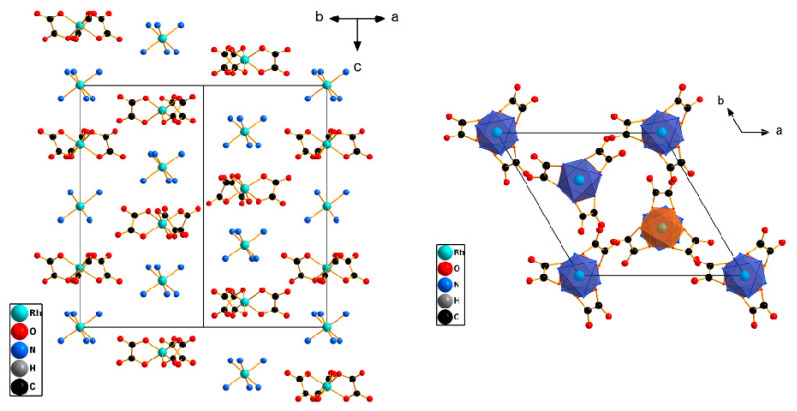
The structure view for the [Rh(NH_3_)_6_][Rh(C_2_O_4_)_3_]·3H_2_O structure, with a side view of the columns. H atoms and water molecules are omitted for clarity.

These structures are formed by the columns of two types, with alternating cation–anion particles directed along the *c* axis (Figure 3). The columns of the first type, oriented along the *c* axis (0, 0, *z*), are formed due to the formation of hydrogen bonds between the ligands of the [M^1^(NH_3_)_6_]^3+^ and [M^2^(C_2_O_4_)_3_]^3−^ complexes. The second type of columns are formed along the *c* axis, (2/3, 1/3, *z*) and (1/3, 2/3, *z*). The distance between metal atoms along the *c* axis is within 4 to 6 Å. The cation and anion are linked by a hydrogen bond, directly between the ligand atoms and between the ligand and water atoms. These columns are interconnected by a system of hydrogen bonds.

Each complex ion is surrounded by eight neighbouring counter-ions. In this case, the neighbours located along the *c* axis have the smallest distances, of about 5 Å, and the remaining distances, M^1^–M^2^, have an average distance of 7.4 to 7.6 Å. These structures have a CsCl structural motif with trigonal distortion.

#### 2.3.3. The Crystal Structure of K_3_[Rh(NH_3_)_6_][Rh(C_2_O_4_)_3_]_2_·6H_2_O

The structure is isolated, consisting of the separate [Rh(NH_3_)_6_]^3+^ and K^+^ cations and [Rh(C_2_O_4_)_3_]^3−^ anions and water molecules. The [Rh(NH_3_)_6_]^3+^ complex ions have Rh-N bond lengths of 2.029(3) Å, corresponding to the same ions in other structures, and the N-Rh-N angles are 89.61(12)–90.39(12)°, which correspond to octahedral angles with a maximum deviation of 0.4°. Cations are at the special point of −3. Both cationic and anionic complexes have the octahedral coordination spheres.

The [Rh(C_2_O_4_)_3_]^3−^ complex anions are propeller-like and have Co-O bond lengths of 2.003(2) and 2.020(2) Å, with an average length of 2.012(2) Å and O-Co-O chiral angles of 83.34(8)–87.71(9). These geometric parameters of the anion correspond to other parameters of the same anions in other structures. The Rh atom is located on axis 3.

There are two types of potassium atoms in the K_3_[Rh(NH_3_)_6_][Rh(C_2_O_4_)_3_]_2_·6H_2_O structure: the first type is located in the position (0, 0, 0) with the symmetry −3 and surrounded by 6 oxalate O atoms (K…O distance is 2.693(2) Å), while the second type is located in the position (0, 0, z) with the symmetry 3 and surrounded by 9 oxalate O atoms (K…O distance varies in the range of 2.705(2)–2.903(2) Å, average length of 2.82(3) Å).

**Figure 4:**
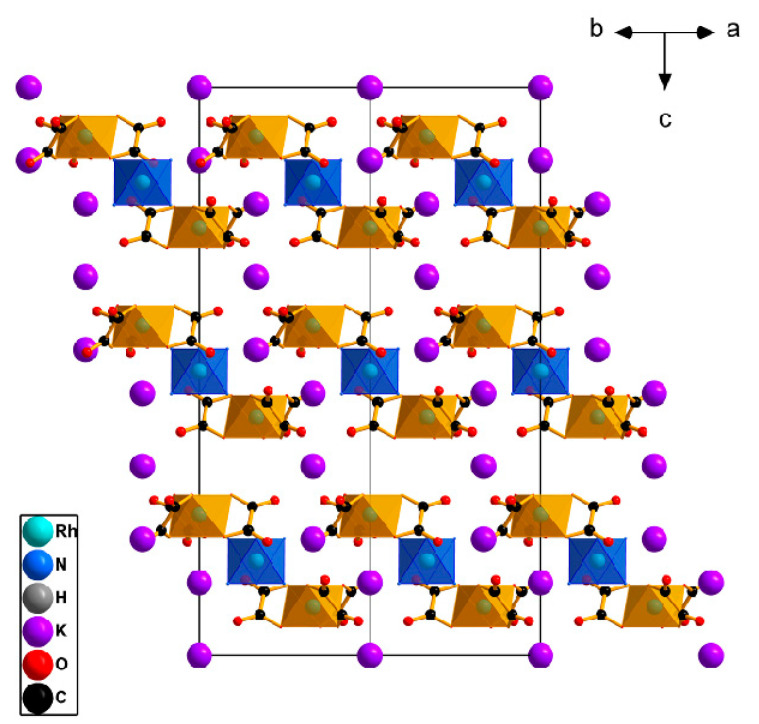
The structure view for K_3_[Rh(NH_3_)_6_][Rh(C_2_O_4_)_3_]_2_·6H_2_O. H atoms and water molecules are omitted for clarity.

The pseudo-layers perpendicular to the *c* axis and formed by [Rh(C_2_O_4_)_3_]^3−^ anions and potassium and [Rh(NH_3_)_6_]^3+^ cations (Figure 4) could be separated in the crystal structure. The cationic layers are divided into two types: the first layer is filled with K^+^ and [Rh(NH_3_)_6_]^3+^, and the second is filled only with K^+^. The distances between anionic layers are 5.02 and 4.73 Å. With sequential stacking, the layers are shifted towards each other by 1/3 of the vector [1 −1 −1].

These layers are stabilised by hydrogen bonds between the ligands of the complexes, as well as between the ligands and water.

### 2.4. Thermal Decomposition of Complex Salts

#### 2.4.1. The Decomposition of [Rh(NH_3_)_6_][Rh(C_2_O_4_)_3_] in Inert and Reducing Atmospheres

**Figure 5:**
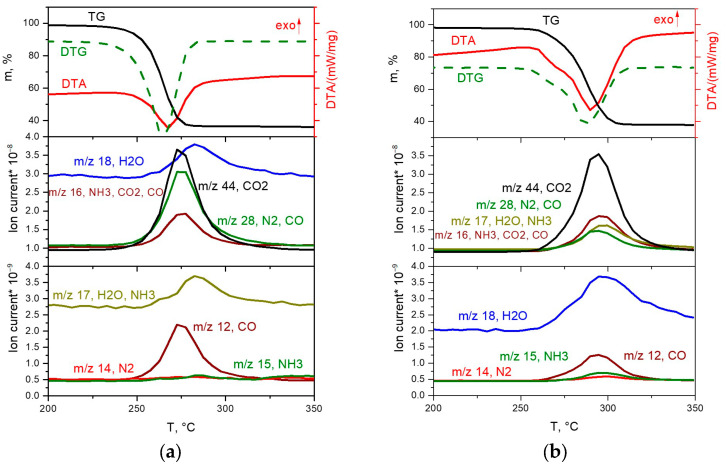
TGA (black line), DTA (red line) and EGA-MS curves for the decomposition of [Rh(NH_3_)_6_][Rh(C_2_O_4_)_3_] ((**a**) in a hydrogen atmosphere, (**b**) in a helium atmosphere).

The thermal decomposition of the [Rh(NH_3_)_6_][Rh(C_2_O_4_)_3_] complex in an inert atmosphere (Figure 5b) takes place in the temperature range of 245–315 °C through two poorly resolved stages, accompanied by endothermic effects. The main gaseous products are CO_2_, CO, NH_3_ and nitrogen. The total mass loss is 62.0%, and the mass of the final product is 38%. The overestimation of the mass of the final product compared to the theoretical value (35.98%) can be explained by the formation of insignificant amounts of amorphous carbon during thermolysis in an inert atmosphere. According to XRD, the final product of thermolysis at 600 °C is metallic rhodium (sp. gr. *F*m-3m, *a* = 3.802 Å, *V*/*Z* = 13.7 Å^3^, crystallite size 5–6 nm):

[Rh(NH_3_)_6_][Rh(C_2_O_4_)_3_] → 2Rh +3CO + 3CO_2_ + N_2_ + 4NH_3_ + 3H_2_O.

In the reducing atmosphere, the complete decomposition of the [Rh(NH_3_)_6_][Rh(C_2_O_4_)_3_] complex (Figure 5a) occurs at a lower temperature and proceeds in the temperature range of 220–290 °C. The process of thermolysis, similar to the inert atmosphere, occurs through two poorly resolved stages, accompanied by endothermic effects. According to mass spectrometry, the main gaseous products are CO_2_, CO, N_2_ and H_2_O. A slight mass loss of ~1% is observed above 290 °C. The total mass loss is 64.4%, and the mass of the final product is 35.6%, which is in good agreement with the theoretical content of rhodium in the initial DCS (35.98%). According to XRD, the final product of thermolysis at 350 °C is metallic rhodium (sp. gr. *F*m-3m, *a* = 3.802 Å, *V*/*Z* = 13.7 Å^3^, crystallite size 7–11 nm):

[Rh(NH_3_)_6_][Rh(C_2_O_4_)_3_] + 3H_2_ → 2Rh + 3CO + 3CO_2_ + 6NH_3_ + 3H_2_O.

#### 2.4.2. The Decomposition of [Co(NH_3_)_6_][Rh(C_2_O_4_)_3_] in Inert and Reducing Atmospheres

**Figure 6:**
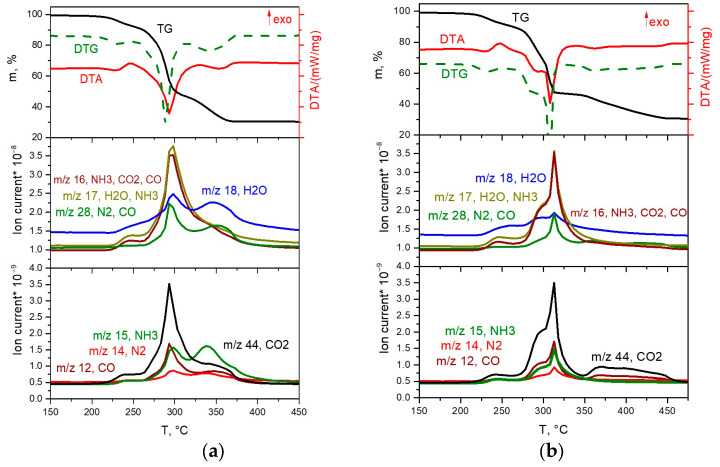
TGA (black line), DTA (red line) and EGA-MS curves for the decomposition of [Co(NH_3_)_6_][Rh(C_2_O_4_)_3_] ((**a**) in a hydrogen atmosphere, (**b**) in a helium atmosphere).

The thermal decomposition of the [Co(NH_3_)_6_][Rh(C_2_O_4_)_3_] complex salt in inert and reducing atmospheres occurs in a similar manner and proceeds in three stages (Figure 6a,b).

The first stage occurs in the temperature range of 200–260 °C, accompanied by an endothermic effect. In the mass spectrum of gaseous products, a significant increase in ionic currents from *m*/*z* = 18, 17, 16 and 15 (H_2_O and NH_3_), and a slight increase in ionic currents from *m*/*z* = 44, 28 and 14 (CO_2_ and N_2_) are observed. According to XRD data, the diffraction pattern of the intermediate product obtained at a temperature of 260 °C contains reflexes related to the CoC_2_O_4_∙nH_2_O phases. Based on the above data, it can be assumed that the first stage is the removal of three ammonia molecules with simultaneous reduction of Co(III) to Co(II), and the formation of the CoC_2_O_4_∙NH_3_ phase and some other phases, with the gross formula of “Rh(NH_3_)_x_(C_2_O_4_)_y_”. Note that the formation of the CoC_2_O_4_∙NH_3_ phase was also observed by the authors of [17,18] in their works devoted to the thermal decomposition of [Co(NH_3_)_6_]_2_(C_2_O_4_)_3_∙4H_2_O. The mass loss at this stage is ~9.5%, which is in good agreement with the calculated value (9.6%). The scheme of this stage is as follows:

[Co(NH_3_)_6_][Rh(C_2_O_4_)_3_] → ”CoC_2_O_4_∙NH_3_” + “Rh(NH_3_)_x_(C_2_O_4_)_y_” + 3NH_3._

**Figure 7:**
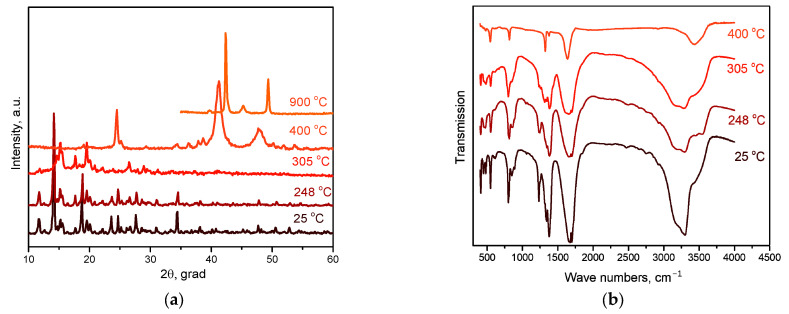
(**a**) XRD patterns of [Co(NH_3_)_6_][Rh(C_2_O_4_)_3_] and the products of its thermolysis in the hydrogen atmosphere at different temperatures. (**b**) IR spectra of [Co(NH_3_)_6_][Rh(C_2_O_4_)_3_] and the products of its thermolysis in the hydrogen atmosphere at different temperatures.

At the second stage of decomposition in the temperature range of 260–310 °C, further decomposition of the intermediate product formed at the first stage occurs. According to mass spectrometry, the main gaseous products are NH_3_, N_2_, CO, CO_2_ and H_2_O. IR spectrometric data of intermediate products obtained at different temperatures (Figure 7b) indicate that the peaks related to the vibrations of ammonia molecules (1334 cm^−1^ δ_s_(HNH), 3300 cm^−1^ ν_a_(NH_3_), 3147 cm^−1^ ν_s_(NH_3_), 849 cm^−1^ ρ_r_(NH_3_) and 449 cm^−1^ ν(CoN)), as well as those related to the vibrations of Rh-O (810, 800, 482 cm^−1^ ν(Rh-O)), decrease with the increasing final heating temperature. In the IR spectrum of the product obtained in the second stage at a temperature of 305 °C, only vibrations related to oxalate vibrations (1638 cm^−1^ ν_a_(CO), 1379, 1321 cm^−1^ ν_s_(CO), 818 cm^−1^ δ(O–C=O)) and Co-O vibrations (484 cm^−1^ ν(Co–O)) are observed, which indicates the complete decomposition of the “Rh(NH_3_)_3_(C_2_O_4_)_1.5_” intermediate product. According to XRD (Figure 7a), the product obtained at a temperature of 305 °C contains CoC_2_O_4_ and an amorphous phase. The total mass loss at the first and second stages is ~53%, and the mass of the intermediate product is 47%. Theoretical calculations yield a 52.7% mass loss, which is in good agreement with the experimental data. The equations of the process reaction are:

CoC_2_O_4_∙NH_3_ → CoC_2_O_4_ + NH_3_,

3“Rh(NH_3_)_x_(C_2_O_4_)_y_” → 3Rh + 3yCO + 3yCO_2_ + yN_2_ + (3x − 2y)NH_3_ + 3yH_2_O.

The third stage of decomposition in reducing and inert atmospheres occurs in the temperature ranges of 310–375 °C and 310–460 °C, respectively. According to mass spectrometry, the main gaseous products are CO and CO_2_. The mass of the final product is 30.6%, which is consistent with the theoretical content of metals (30.65%) in the initial DCS. The equation of the process reaction is:

Rh + CoC_2_O_4_ → 2Co_0.5_Rh_0.5_ + 2CO_2_.

The final decomposition product obtained in the hydrogen atmosphere at 500 °C, according to XRD data, is a mixture of 46.6% face-centred cubic (FCC) phase (sp. gr. *P*6_3_/mmc, *a* = 2.630, *c* = 4.243 Å, *V*/*Z* = 12.7 Å^3^, Co_0.46_Rh_0.54_, crystallite size 12–15 nm) and 53.4% hexagonal close-packed (HCP) phase (sp. gr. *F*m-3m, *a* = 3.709 Å, *V*/*Z* = 12.8 Å^3^, Co_0.44_Rh_0.56_, crystallite size 7–9 nm), resulting in FCC phase (sp. gr. *F*m-3m, *a* = 3.694 Å, *V*/*Z* = 12.6 Å^3^, Co_0.5_Rh_0.5_, crystallite size 15–42 nm) upon further heating up to 800 °C. The final decomposition product obtained in the helium atmosphere at 900 °C is a mixture of 15.0% HCP phase (sp. gr. *P*6_3_/mmc, *a* = 2.619, *c* = 4.256 Å, *V*/*Z* = 12.7 Å^3^, Co_0.48_Rh_0.52_, crystallite size 12–16 nm) and 85.0% FCC phase (sp. gr. *F*m-3m, *a* = 3.693 Å, *V*/*Z* = 12.6 Å^3^, Co_0.5_Rh_0.5_, crystallite size 29–43 nm).

#### 2.4.3. The Decomposition of [Rh(NH_3_)_6_][Co(C_2_O_4_)_3_] in Inert and Reducing Atmospheres

**Figure 8:**
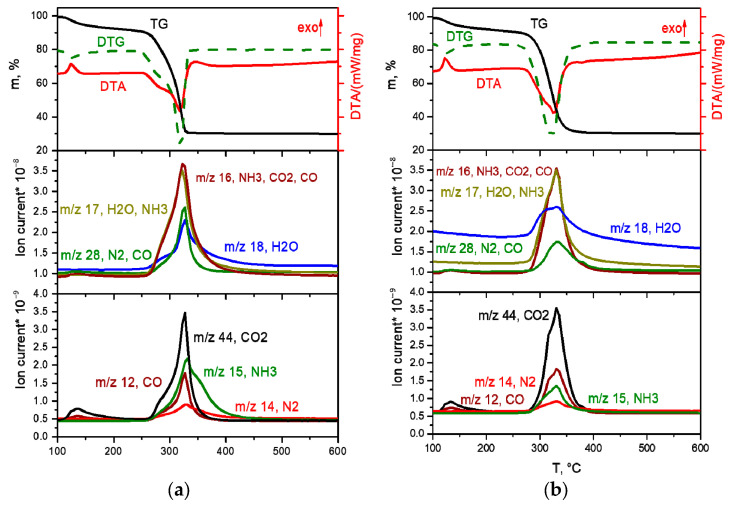
TGA (black line), DTA (red line) and EGA-MS curves for the decomposition of [Rh(NH_3_)_6_][Co(C_2_O_4_)_3_] ((**a**) in a hydrogen atmosphere, (**b**) in a helium atmosphere).

The decomposition of the [Rh(NH_3_)_6_][Co(C_2_O_4_)_3_] complex salt in inert and reducing atmospheres is almost identical (Figure 8a,b). The main difference is the lower temperature of the end of the thermolysis process in the reducing atmosphere (340 °C) compared to the inert (380 °C), as well as the release of greater amounts of ammonia at the third stage of decomposition. Let us consider in detail the decomposition of the complex in a reducing atmosphere.

**Figure 9:**
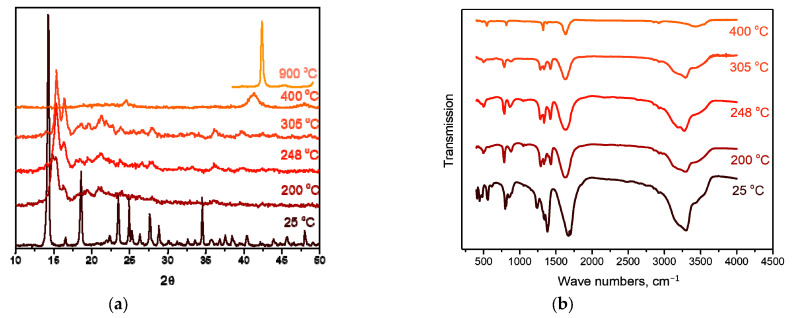
(**a**) XRD patterns of [Rh(NH_3_)_6_][Co(C_2_O_4_)_3_] and the products of its thermolysis in the hydrogen atmosphere at different temperatures. (**b**) IR spectra of [Rh(NH_3_)_6_][Co(C_2_O_4_)_3_] and the products of its thermolysis in the hydrogen atmosphere at different temperatures.

The decomposition of the complex proceeds in three stages. The first stage, in the temperature range of 100–150 °C, is accompanied by a pronounced exothermic effect and leads to a mass loss of ~5.5%. The main gaseous product is CO_2_. According to XRD, the intermediate product obtained at a temperature of 200 °C is a mixture of cobalt oxalate and the amorphous phase (Figure 9a). In this case, the IR spectrum of the intermediate product obtained at this temperature features the bands related to the vibrations of the [Rh(NH_3_)_6_]^3+^ cation, as well as CoC_2_O_4_-related vibrations. Therefore, cobalt(II)-oxalate and the intermediate “[Rh(NH_3_)_6_](C_2_O_4_)_1.5_” product can be assumed when decomposing [Rh(NH_3_)_6_][Co(C_2_O_4_)_3_]. The equation of the process reaction is:

2[Rh(NH_3_)_6_][Co(C_2_O_4_)_3_] → “[Rh(NH_3_)_6_]_2_(C_2_O_4_)_3_” + 2CoC_2_O_4_ + 2CO_2_.

The second and third stages are poorly resolved, they occur in the temperature range of 150–340 °C and are accompanied by endothermic effects. The main gaseous products are NH_3_, H_2_O, CO, CO_2_ and N_2_. The analysis of the IR spectra of intermediate products obtained at different temperatures indicates that with an increase in temperature, the intensity of all vibrational bands decreases. In this case, the vibrations related to the [Rh(NH_3_)_6_]^3+^ cation are present until almost the end of thermolysis (Figure 9b). Therefore, a decomposition of “[Rh(NH_3_)_6_]_2_(C_2_O_4_)_3_” to metallic rhodium can be assumed at the beginning of the stage. With subsequent heating, the decomposition of CoC_2_O_4_ to cobalt occurs, and its introduction into the rhodium lattice takes place with the formation of Co_x_Rh_1−x_ solid solution. This assumption is confirmed by XRD data, since at 325 °C the reflexes corresponding to the CoC_2_O_4_ phase and the Co_0.15_Rh_0.85_ solid solution (sp. gr. *F*m-3m, *a* = 3.776 Å, *V*/*Z* = 13.5 Å^3^, crystallite size 4–5 nm) containing a greater amount of rhodium are observed in the diffraction patterns. Then, it can be assumed that:

2“[Rh(NH_3_)_6_]_2_(C_2_O_4_)_3_” → 2Rh + N_2_ + 10NH_3_ + 3CO_2_ + 3CO + 3H_2_O,

The final decomposition product in the helium atmosphere at 900 °C is a mixture of FCC phase (sp. gr. *F*m-3m, *a* = 3.693 Å, *V*/*Z* = 12.6 Å^3^, Co_0.5_Rh_0.5_, crystallite size 17–24 nm) and a small amount of HCP phase. The final decomposition product in the hydrogen atmosphere at 800 °C is a mixture of 14.7% HCP phase (sp. gr. *P*6_3_/mmc, *a* = 2.631, *c* = 4.209 Å, *V*/*Z* = 12.6 Å^3^, Co_0.5_Rh_0.5_, crystallite size 4–5 nm) and 85.3% FCC phase (sp. gr. *F*m-3m, *a* = 3.692 Å, *V*/*Z* = 12.6 Å^3^, Co_0.5_Rh_0.5_, crystallite size 17–24 nm).

#### 2.4.4. The Decomposition of K_3_[Rh(NH_3_)_6_][Rh(C_2_O_4_)_3_]_2_·6H_2_O in Inert and Reducing Atmospheres

**Figure 10:**
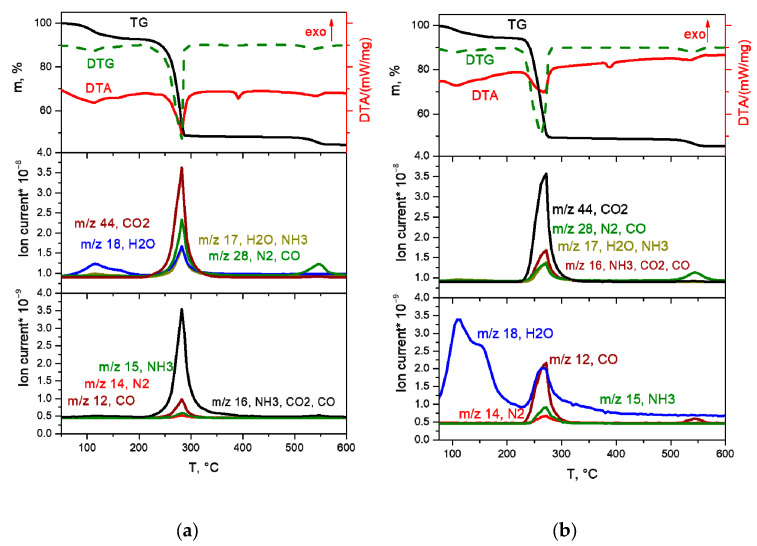
TGA curves (black line), DTA (red line) and EGA-MS for the decomposition of K_3_[Rh(NH_3_)_6_][Rh(C_2_O_4_)_3_]_2_·6H_2_O ((**a**) in a hydrogen atmosphere, (**b**) in a helium atmosphere).

K_3_[Rh(NH_3_)_6_][Rh(C_2_O_4_)_3_]_2_·6H_2_O salt decomposition in inert and reducing atmospheres is generally identical (Figure 10a,b).

The first stage of decomposition—the removal of crystallisation water molecules—occurs in the temperature range of 90–180 °C and proceeds in several stages:

K_3_[Rh(NH_3_)_6_][Rh(C_2_O_4_)_3_]_2_·6H_2_O → K_3_[Rh(NH_3_)_6_][Rh(C_2_O_4_)_3_]_2_ + 6H_2_O.

The mass loss at this stage is ~9.0%, which is in agreement with the calculated value (9.3%).

At the next stage, in the temperature range of 200–280 °C, the decomposition of the anhydrous complex occurs with the formation of metallic rhodium and K_2_C_2_O_4_. The decomposition process takes place in the same temperature range as the DCS [Rh(NH_3_)_6_][Rh(C_2_O_4_)_3_] decomposition. The main gaseous products are CO_2_, CO, NH_3_ and H_2_O:

2K_3_[Rh(NH_3_)_6_][Rh(C_2_O_4_)_3_]_2_ → 6Rh + 3K_2_C_2_O_4_ + 6CO + 12CO_2_ + 6H_2_O + 2N_2_ + 8NH_3_.

Note that the decomposition of the complex occurs in the same temperature range as that for [Rh(NH_3_)_6_][Rh(C_2_O_4_)_3_], with the release of the same gaseous products. However, due to the presence of potassium in the initial DCS, the final product, in addition to metallic rhodium, contains K_2_C_2_O_4_. At a temperature of 382 °C, an endothermic effect is observed on the DTA curve caused by the K_2_C_2_O_4_ melting (according to the literature, the melting temperature of K_2_C_2_O_4_ is 397 °C [19]). At the last decomposition stage at 500–570 °C, the K_2_C_2_O_4_ decomposition occurs to K_2_CO_3_ and CO (according to literature, the decomposition temperature of K_2_C_2_O_4_ is over 500 °C [19]). The final product obtained at a temperature of 600 °C, according to XRD, is a mixture of metal Rh and K_2_CO_3_.

The thermal decomposition of the synthesised complex salts in helium and hydrogen atmospheres is quite similar, with the exception that in a reducing atmosphere, the temperatures of the beginning and end of the processes are slightly less. This is because hydrogen is also involved in the reduction of metals. After the dehydration process, the decomposition of [Rh(NH_3_)_6_][Rh(C_2_O_4_)_3_] proceeds in almost one stage, in contrast to [Co(NH_3_)_6_][Rh(C_2_O_4_)_3_] and [Rh(NH_3_)_6_][Co(C_2_O_4_)_3_], which decompose with the formation of intermediate products: CoC_2_O_4_ and X-ray amorphous rhodium compounds. Due to the formation of the intermediate product CoC_2_O_4_, the range of temperatures for the decomposition of the DCS increases by approximately 150 °C. The decomposition of [Rh(NH_3_)_6_][Rh(C_2_O_4_)_3_] leads to the formation of metallic Rh, while [Co(NH_3_)_6_][Rh(C_2_O_4_)_3_] and [Rh(NH_3_)_6_][Co(C_2_O_4_)_3_] decompose with the formation of a mixture of Co_x_Rh_1-x_ solid solutions based on HCP and FCC lattices. Upon further heating, this mixture transforms into a Co_0.50_Rh_0.50_ solid solution based on the FCC lattice.

## 3. Materials and Methods

### 3.1. The Synthesis of Compounds

The initial compounds, [Co(NH_3_)_6_]Cl_3_, [Rh(NH_3_)_6_]Cl_3_, (NH_4_)_3_[Co(C_2_O_4_)_3_] and K_3_[Rh(C_2_O_4_)_3_], were synthesised according to well-known techniques [15,20,21,22]. The identification and single-phase composition of the initial compounds were confirmed by the SCXRD using the PDF2 database or the known monocrystalline data.

#### 3.1.1. The Synthesis of [Rh(NH_3_)_6_][Rh(C_2_O_4_)_3_]

Weighed amounts of [Rh(NH_3_)_6_]Cl_3_ (0.0453 g, 0.1461 mmol) and K_3_[Rh(C_2_O_4_)_3_] (0.0704 g, 0.1455 mmol), in a molar ratio of 1:1, were dissolved in a minimum amount of water (3 mL and 5 mL, respectively). The resulting solutions were mixed, and immediately a pale-orange precipitate was obtained. The solution with the precipitate was boiled for 30 min, while the colour of the precipitate passed from a pale orange to a saturated orange colour. The precipitate solution was cooled to room temperature, filtered on a glass filter, washed with a small amount of water and dried in the air.

It should be noted that during synthesis at room temperature, a mixture was formed comprising [Rh(NH_3_)_6_][Rh(C_2_O_4_)_3_] and K_3_[Rh(NH_3_)_6_][Rh(C_2_O_4_)_3_]_2_∙6H_2_O in an approximate ratio of 1:1.

The yield was 82%. The compound is a fine-crystalline powder of orange colour.

Anal. Calc./Found for C_6_H_18_N_6_O_12_Rh_2_: C, 12.6/12.0%; H, 3.2/3.4%; N, 14.7/14.2%; Rh, 36.0/35.7%.

Selected IR spectral data (KBr, cm^−1^): 3298b, 3184b, 2479vs, 1680b, 1383b, 1329b, 1233m, 889s, 853m, 800b, 617vs, 546m, 484s, 453s, 409m.

When trying to grow a single crystal of this salt by slowly evaporating the mother liquor in air, only the hydrate single crystals of the [Rh(NH_3_)_6_][Rh(C_2_O_4_)_3_]·3H_2_O composition, as well as the monocrystals of the K_3_[Rh(NH_3_)_6_][Rh(C_2_O_4_)_3_]_2_∙6H_2_O composition, were obtained.

#### 3.1.2. The Synthesis of [Rh(NH_3_)_6_][Co(C_2_O_4_)_3_] and [Rh(NH_3_)_6_][Co(C_2_O_4_)_3_]·3H_2_O

The synthesis of [Rh(NH_3_)_6_][Co(C_2_O_4_)_3_] was carried out during boiling, similar to the synthesis of [Rh(NH_3_)_6_][Rh(C_2_O_4_)_3_] (Section 3.1.1.), wherein [Rh(NH_3_)_6_]Cl_3_ and (NH_4_)_3_[Co(C_2_O_4_)_3_] complex salts were taken as starting compounds. In contrast, for the synthesis of [Rh(NH_3_)_6_][Co(C_2_O_4_)_3_]·3H_2_O, the precipitate solution was stirred at room temperature.

The yield of [Rh(NH_3_)_6_][Co(C_2_O_4_)_3_] was 75%. The compound is a fine-crystalline powder of blue-green colour.

Anal. Calc./Found for C_6_CoH_18_N_6_O_12_Rh: C, 13.6/13.8%; H, 3.4/3.3%; N, 15.9/15.3%; (Co, Rh), 30.7/30.2%.

Selected IR spectral data (KBr, cm^−1^): 3495m, 3292b, 3146b, 2496vs, 1711m, 1649b, 1391b, 1341b, 1314m, 1246m, 893s, 860m, 797b, 621vs, 561m, 486s, 469s, 446m.

The yield of [Rh(NH_3_)_6_][Co(C_2_O_4_)_3_]·3H_2_O was 80%. The compound is a fine-crystalline powder of blue-green colour.

Anal. Calc./Found for C_6_CoH_24_N_6_O_15_Rh: C, 12.4/12.4%; H, 4.1/4.2%; N, 14.4/14.1%.

Selected IR spectral data (KBr, cm^−1^): 3545m, 3455m, 3312b, 3188b, 2922vs, 2464vs, 2509vs, 2367vs, 2199vs, 1686b, 1400b, 1342m, 1256m, 874s, 820m, 567m, 473s, 447m.

The single crystals of both compounds were obtained by slow counter diffusion through agar–agar. It is worth noting that anhydrous salt and hydrate were formed in one experiment, where the colour of the anhydrous salt crystals was blue, and that of the hydrate was green.

#### 3.1.3. The Synthesis of [Co(NH_3_)_6_][Rh(C_2_O_4_)_3_]

The synthesis of [Co(NH_3_)_6_][Rh(C_2_O_4_)_3_] was carried out similarly to the synthesis of [Rh(NH_3_)_6_][Rh(C_2_O_4_)_3_], wherein the [Co(NH_3_)_6_]Cl_3_ and K_3_[Rh(C_2_O_4_)_3_] complex salts were taken as starting compounds.

The product yield was 89%. The compound is a fine-crystalline powder of yellow colour.

Anal. Calc./Found for C_6_CoH_18_N_6_O_12_Rh: C, 13.65/13.7%; H, 3.44/3.4%; N, 15.91/15.4%; (Co, Rh), 30.65/29.5%.

Selected IR spectral data (KBr, cm^−1^): 3306b, 3163b, 2749vs, 2461vs, 1703b, 1375b, 1346b, 1233m, 887s, 851m, 799b, 613vs, 546m, 453s, 411m.

The single crystals of [Co(NH_3_)_6_][Rh(C_2_O_4_)_3_]·3H_2_O were obtained by slow counter diffusion through agar–agar.

#### 3.1.4. The Synthesis of K_3_[Rh(NH_3_)_6_][Rh(C_2_O_4_)_3_]_2_·6H_2_O

To obtain the complex compound, the weighed amounts of [Rh(NH_3_)_6_]Cl_3_ (0.0295 g, 0.0947 mmol) and K_3_[Rh(C_2_O_4_)_3_] (0.0917 g, 0.1896 mmol) were taken in a molar ratio of 1:2, respectively, and dissolved separately in 7 mL of water each. The solutions were mixed, and the resulting precipitate solution was left for 1 h at room temperature. After that, the precipitate was filtered and washed with a small amount of water.

The yield was 76%. The compound is a fine-crystalline powder of yellow colour.

Anal. Calc./Found for C_12_H_30_K_3_N_6_O_21_Rh_3_: C, 12.38/12.5%; H, 2.60/2.6%; N, 7.22/7.4%; (Rh + 3/2K_2_CO_3_), 44.32/44.5%.

Selected IR spectral data (KBr, cm^−1^): 3780b, 3501b, 3296b, 2913vs, 2799vs, 2641vs, 2498vs, 1659b,1396b, 1341m, 1246m, 897s, 874s, 806b, 554m, 573m, 407m.

The single crystals of K_3_[Rh(NH_3_)_6_][Rh(C_2_O_4_)_3_]_2_∙6H_2_O were obtained by slowly evaporating the mother liquor after the synthesis of [Rh(NH_3_)_6_][Rh(C_2_O_4_)_3_] salt in air, as indicated above.

### 3.2. Characterisation

X-ray diffraction analysis of the samples was performed on a DRON-RM4 diffractometer (Cu-Kα radiation, a graphite monochromator using a reflected beam and a scintillation detector with amplitude discrimination, Brevetting, Saint Petersburg, Russia). The samples were prepared by applying a suspension in alcohol on the polished side of a fused quartz cuvette. A polycrystalline silicon sample (*a* = 5.4309 Å) prepared in the same way was used as an external reference sample. The diffraction patterns were recorded in a step-by-step mode in a *2θ* angles range of 5°–120°.

X-ray phase analysis (XRD) of the thermolysis products was carried out in accordance with the data provided in the PDF file for pure substances [23]. Parameters of the metal phases were refined over the entire data array using the Powder Cell 2.4 application program [24]. The size of the crystallites in the resulting metal powders was estimated from the coherent scattering regions as a result of Fourier analysis of the profiles of single diffraction peaks using the WINFIT 1.2.1 software [25].

The composition of the obtained bimetallic solid solutions was determined using calibration curves of the volume per atom ratio (*V*/*Z*, where *V* is the volume of the unit cell, and *Z* is the number of structural units in it—atoms, in this case) depending on the concentration of one of the metals. The calibration curves were plotted from the experimental values of atomic volumes for single-phase solid solutions of known composition, provided in the references for Co-Rh systems [26,27,28,29].

The single-crystal X-ray diffraction data for 2277194 and 2277196 were collected on a X8APEX Bruker Nonius diffractometer, equipped with a 4K CCD area detector, and for 2277197, 2277195 and 2277193 on a Bruker D8 Venture diffractometer with a CMOS PHOTON III detector and an IµS 3.0 source (Montel mirror optics), using the graphite monochromatized MoKα radiation (*λ*= 0.71073 Å) at 150(2) K (Table 2). The *θ*- and ω-scan techniques were employed to measure the intensities. Absorption corrections were empirically applied using the SADABS program [30]. Structures were solved by the direct methods of the difference Fourier synthesis and further refined by the full-matrix least squares method using the SHELXTL package [31]. Atomic thermal parameters for non-hydrogen atoms were anisotropically refined. The positions of hydrogen atoms of amino groups were calculated corresponding to their geometrical conditions and refined using the riding model, while the water molecule’s hydrogen atoms were refined in the isotropic approximation, with the distance constraint of about 0.96 Å, or unlocalised.

For IR and elemental analysis, IR spectra were recorded on a Bruker Vertex 80V FTIR spectrometer in the range of 4000–400 cm^−1^ from pellets pressed with KBr. The attribution of IR spectral bands was conducted by comparison with the literature data [16].

Elemental (CHN) analysis was carried out with a Euro EA 3000 analyser. To analyse the metal content in the salts, weighted samples of the DCS (~50 mg) were placed in a tubular quartz reactor. Heating was performed in a split furnace at the rate of 10 K/min in a hydrogen atmosphere. After reaching the final temperature, samples were kept for 1 h, and then the hydrogen stream was switched off and the system was purged with helium for 0.5 h. Afterwards, the furnace was removed, and the reactor was allowed to cool to ambient temperature in a continuous helium stream. The product was then weighed.

For thermal analysis, the simultaneous TG–DTA/EGA-MS measurement was performed using an STA 449 F1 Jupiter thermal analyser connected to an QMS 403D Aëolos quadrupole mass spectrometer (NETZSCH, Selb, Germany). The spectrometer was connected online to a thermal analyser (STA) by a quartz capillary heated to 280 °C. The QMS was operated with an electron impact ioniser with the energy of 70 eV. The ion currents of the selected mass/charge (*m*/*z*) numbers were monitored in multiple-ion detection (MID) mode with the collection time of 0.1 s for each channel. The measurements were carried out in a helium–hydrogen mixture (10.0 vol.% H_2_) or helium atmosphere in the temperature range of 30–500 °C, using the heating rate of 10 °C min^−1^, the gas flow rate of 30 mL min^−1^ and opened Al_2_O_3_ crucibles.

The processing of the experimental results was performed using the conventional Proteus Analysis software v.6.1.0 [32].

## 4. Conclusions

This paper presented the methods of synthesis of [M^1^(NH_3_)_6_][M^2^(C_2_O_4_)_3_] (M^1^, M^2^ = Co, Rh) double-complex salts and the studies of their crystalline structure. It was shown that varying the synthesis temperature allowed to obtain isostructural compounds in the “cobalt-rhodium” rows, which in turn led to the possibility of the synthesis of continuous series of solid solutions based on double-complex salts in the [Rh(NH_3_)_6_][Rh(C_2_O_4_)_3_]—[Rh(NH_3_)_6_][Co(C_2_O_4_)_3_] and [Rh(NH_3_)_6_][Rh(C_2_O_4_)_3_]—[Co(NH_3_)_6_][Rh(C_2_O_4_)_3_] rows-nanoalloy precursors. The synthesis at room temperature resulted in the formation of the K_3_[Rh(NH_3_)_6_][Rh(C_2_O_4_)_3_]_2_∙6H_2_O structure, which was not formed during boiling. Thermal properties in inert and reducing atmospheres were studied in detail, and a staged thermolysis mechanism was proposed. It was shown that complete decomposition of complex salts to metals or solid solutions was achieved at temperatures of 320–450 °C.

## Figures and Tables

**Table 1:** Geometric parameters of complex compounds of the type [M_1_(NH_3_)_6_][M_2_(C_2_O_4_)_3_]·3H_2_O.

	[Rh(NH_3_)_6_][Rh(C_2_O_4_)_3_] 3H_2_O	[Rh(NH_3_)_6_][Co(C_2_O_4_)_3_] 3H_2_O	[Co(NH_3_)_6_][Rh(C_2_O_4_)_3_] 3H_2_O
M_1_-N, Å	2.0631(17)–2.0809(16)	2.061(3)–2.076(2)	1.955(4)–1.972(5)
Average, Å	2.0731(12)	2.0705(10)	1.9636(10)
N-M_1_-N, °	87.44(7)–91.50(7)	88.13(13)–91.37(13)	86.80(18)–92.47(19)
M_2_–O, Å	2.0037(14)–2.0191(14)	1.884(2)–1.910(2)	1.9981(12)–2.0131(13)
Average, Å	2.0113(13)	1.896(3)	2.0072(11)
O-M_2_-O, °	82.85(6)–83.39(6)	85.57(10)–86.26(11)	83.10(6)–86.24(5)

**Table 2:** Crystal data and structure refinement.

CCDC Number	2277193	2277194	2277195	2277196	2277197
Empirical formula	[Rh(NH_3_)_6_] [Rh(C_2_O_4_)_3_]·3H_2_O	[Rh(NH_3_)_6_] [Co(C_2_O_4_)_3_]·3H_2_O	[Co(NH_3_)_6_] [Rh(C_2_O_4_)_3_]·3H_2_O	[Rh(NH_3_)_6_] [Co(C_2_O_4_)_3_]	K_3_[Rh(NH_3_)_6_] [Rh(C_2_O_4_)_3_]_2_·6H_2_O
Formula weight	626.13	582.15	582.15	528.10	1164.45
Temperature	150(2) K	293(2) K	150(2) K	293(2) K	150(2) K
Crystal system	trigonal	trigonal	trigonal	monoclinic	trigonal
Space group	*P*-3	*P*-3	*P*-3	*P*-1	*R*-3
Unit cell dimensions	*a* = 12.5067(3) Å *c* = 21.2408(6) Å	*a* = 12.3686(3) Å *c* = 21.3200(5) Å	*a* = 12.4765(4) Å *c* = 31.1699(17) Å	*a* = 7.5278(2) Å *b* = 9.6146(2) Å *c* = 11.7285(3) Å *α* = 84.827(1)°. *β* = 87.866(1)°. *γ* = 71.567(1)°.	*a* = 10.1393(2) Å *c* = 29.2294(9) Å
Volume	2877.31(16) Å^3^	2824.61(15) Å^3^	4202.0(4) Å^3^	802.00(3) Å^3^	2602.35(13) Å^3^
Z	6	6	9	2	3
F(000)	1872	1764	2646	528	1728
Crystal size	0.15 × 0.05 × 0.04 mm^3^	0.14 × 0.12 × 0.12 mm^3^	0.1 × 0.1 × 0.01 mm^3^	0.14 × 0.12 × 0.12 mm^3^	0.15 × 0.10 × 0.03 mm^3^
Index ranges	−18 ≤ h ≤ 18, −15 ≤ k ≤ 17, −31 ≤ l ≤ 31	−15 ≤ h ≤ 14, −9 ≤ k ≤ 15, −27 ≤ l ≤ 27	−15 ≤ h ≤ 15, −16 ≤ k ≤ 14, −39 ≤ l ≤ 40	−8 ≤ h ≤ 9, −11 ≤ k ≤ 12, −15 ≤ l ≤ 15	−12 ≤ h ≤ 14, −14 ≤ k ≤ 14, −41 ≤ l ≤ 41
Reflections collected	35409	21106	65819	6831	9607
Independent reflections	6208 [R(int) = 0.0356]	4162 [R(int) = 0.0420]	6219 [R(int) = 0.0796]	3649 [R(int) = 0.0271]	1774 [R(int) = 0.0290]
Completeness to θ = 25.250°	99.2%	99.7%	99.7%	98.7%	99.2%
Data/restraints/parameters	6208/0/269	4162/5/286	6219/0/324	3649/0/241	1774/0/95
Goodness-of-fit on F2	1.071	1.043	1.101	1.030	1.092
Final R indices [I > 2sigma(I)]	R1 = 0.0295, wR2 = 0.0769	R1 = 0.0304, wR2 = 0.0701	R1 = 0.0630, wR2 = 0.1213	R1 = 0.0276, wR2 = 0.0682	R1 = 0.0356, wR2 = 0.0996
R indices (all data)	R1 = 0.0354, wR2 = 0.0817	R1 = 0.0381, wR2 = 0.0733	R1 = 0.0852, wR2 = 0.1276	R1 = 0.0314, wR2 = 0.0698	R1 = 0.0396, wR2 = 0.1024

## Data Availability

All data generated or analysed during this study are included in this published article.
